# Current Cigarette Smoking Among Adults — United States, 2016

**DOI:** 10.15585/mmwr.mm6702a1

**Published:** 2018-01-19

**Authors:** Ahmed Jamal, Elyse Phillips, Andrea S. Gentzke, David M. Homa, Stephen D. Babb, Brian A. King, Linda J. Neff

**Affiliations:** 1 Office on Smoking and Health, National Center for Chronic Disease Prevention and Health Promotion, CDC.

The U.S. Surgeon General has concluded that the burden of death and disease from tobacco use in the United States is overwhelmingly caused by cigarettes and other combusted tobacco products (*1*). Cigarettes are the most commonly used tobacco product among U.S. adults, and about 480,000 U.S. deaths per year are caused by cigarette smoking and secondhand smoke exposure (*1*). To assess progress toward the *Healthy People 2020* target of reducing the proportion of U.S. adults aged ≥18 years who smoke cigarettes to ≤12.0% (objective TU-1.1),* CDC analyzed data from the 2016 National Health Interview Survey (NHIS). In 2016, the prevalence of current cigarette smoking among adults was 15.5%, which was a significant decline from 2005 (20.9%); however, no significant change has occurred since 2015 (15.1%). In 2016, the prevalence of cigarette smoking was higher among adults who were male, aged 25–64 years, American Indian/Alaska Native or multiracial, had a General Education Development (GED) certificate, lived below the federal poverty level, lived in the Midwest or South, were uninsured or insured through Medicaid, had a disability/limitation, were lesbian, gay, or bisexual (LGB), or had serious psychological distress. During 2005–2016, the percentage of ever smokers who quit smoking increased from 50.8% to 59.0%. Proven population-based interventions are critical to reducing the health and economic burden of smoking-related diseases among U.S. adults, particularly among subpopulations with the highest smoking prevalences (*1*,*2*).

NHIS is an annual, nationally representative in-person survey of the noninstitutionalized U.S. civilian population. The NHIS core questionnaire is administered to a randomly selected adult in the household (the sample adult). In 2016, the NHIS was administered to 33,028 adults aged ≥18 years; the response rate was 54.3%. Current cigarette smokers were respondents who reported having smoked ≥100 cigarettes during their lifetime and were smoking every day or some days at the time of interview. Former smokers were respondents who reported having smoked ≥100 cigarettes during their lifetime but were not smoking at the time of interview. The mean number of cigarettes smoked per day was calculated among daily smokers. Quit ratios were defined as the ratio of former smokers to ever smokers (i.e., persons who had smoked ≥100 cigarettes during their lifetime).

Data were weighted to adjust for differences in the probability of selection and nonresponse and to provide nationally representative estimates. Current smoking was assessed overall and by sex, age, race/ethnicity, education, poverty status,^†^ U.S. Census region,^§^ health insurance coverage at the time of survey,^¶^ disability/limitation status,** sexual orientation,^††^ and presence or absence of serious psychological distress.^§§^ Differences between groups were assessed using a Wald F test, with statistical significance defined as p<0.05. Population counts were estimated from extrapolated probability weights, rounded down to the nearest 10,000 persons. Quit ratios were calculated overall and by age group. Logistic regression was used to assess overall trends in prevalence, cigarettes smoked per day, and quit ratios during 2005–2016, controlling for sex, age, and race/ethnicity. T-tests were performed to examine differences between 2015 and 2016.

In 2016, 15.5% (37.8 million) of U.S. adults were current cigarette smokers (Table). Overall, smoking prevalence did not change significantly from 2015 (15.1%) to 2016 (15.5%). Current cigarette smoking prevalence was higher among males (17.5%) than among females (13.5%). By age group, prevalence was higher among adults aged 25–44 years (17.6%) and 45–64 years (18.0%) than among those aged 18–24 years (13.1%) or ≥65 years (8.8%). Prevalence was highest among American Indian/Alaska Natives (31.8%) and lowest among non-Hispanic Asians (9.0%). Among adults aged ≥25 years, prevalence was highest among persons with a GED (40.6%) and lowest among those with a graduate degree (4.5%). Prevalence was higher among persons living below the poverty level (25.3%) than those at or above this level (14.3%). By region, prevalence was higher in the Midwest (18.5%) and South (16.9%) than the West (12.3%) or Northeast (13.3%). By insurance status, prevalence was higher among Medicaid enrollees (25.3%) and uninsured adults (28.4%) than among those covered by private insurance (11.8%), Medicare only (10.2%), or other public insurance (19.8%). Prevalence was higher among adults with a disability/limitation (21.2%) than among those with no disability/limitation (14.4%). Prevalence was higher among LGB adults (20.5%) than among heterosexual adults (15.3%) and among adults with serious psychological distress (35.8%) than among those without serious psychological distress (14.7%).

**TABLE:** Characteristics of current adult cigarette smokers* — National Health Interview Survey, United States, 2016

Characteristic	Males (n = 14,991)		Females (n = 18,037)		Total (n = 33,028)	
	Weighted % (95% CI)	Population estimate^†^	Weighted % (95% CI)	Population estimate	Weighted % (95% CI)	Population estimate
**Overall**	17.5 (16.6–18.5)	20,660,000	13.5 (12.8–14.3)	17,110,000	15.5 (14.8–16.1)	37,770,000
**Age group (yrs)**						
18–24	14.7 (12.1–17.3)	2,180,000	11.5 (9.4–13.7)	1,700,000	13.1 (11.4–14.8)	3,890,000
25–44	20.6 (19.0–22.3)	8,480,000	14.6 (13.3–15.9)	6,170,000	17.6 (16.5–18.7)	14,660,000
45–64	19.3 (17.9–20.8)	7,820,000	16.8 (15.5–18.0)	7,190,000	18.0 (17.0–19.0)	15,020,000
≥65	10.1 (8.8–11.5)	2,160,000	7.7 (6.7–8.7)	2,030,000	8.8 (8.0–9.6)	4,200,000
**Race/Ethnicity^§^**						
White	17.8 (16.8–18.8)	13,570,000	15.5 (14.6–16.5)	12,530,000	16.6 (15.9–17.4)	26,100,000
Black	20.2 (17.2–23.2)	2,600,000	13.5 (11.5–15.5)	2,130,000	16.5 (14.7–18.3)	4,730,000
Hispanic	14.5 (11.8–17.2)	2,780,000	7.0 (5.6–8.3)	1,350,000	10.7 (9.2–12.3)	4,140,000
AI/AN	29.3 (19.3–39.4)	230,000	34.3 (24.4–44.2)	260,000	31.8 (24.1–39.5)	490,000
Asian^¶^	14.0 (10.7–17.3)	910,000	4.6 (2.8–6.4)	340,000	9.0 (7.1–10.9)	1,260,000
Multirace	27.7 (19.9–35.5)	520,000	22.9 (16.5–29.2)	460,000	25.2 (20.4–30.0)	990,000
Education level**						
0–12 yrs (no diploma)	28.9 (25.7–32.1)	3,760,000	19.5 (17–22)	2,590,000	24.1 (22.1–26.2)	6,360,000
≤8th grade	22.4 (16.9–27.8)	1,100,000	10.4 (7.7–13.1)	530,000	16.2 (13.3–19.2)	1,630,000
9th–11th grade	35.1 (30.4–39.8)	2,070,000	26.2 (22.5–29.8)	1,530,000	30.7 (27.6–33.7)	3,610,000
12th grade (no diploma)	26.7 (20.7–32.8)	580,000	22.8 (14.8–30.9)	520,000	24.8 (19.8–29.7)	1,100,000
GED	45.5 (38.7–52.2)	1,350,000	36.1 (30.1–42.0)	1,140,000	40.6 (36.1–45.1)	2,490,000
High school graduate	23.1 (21.1–25.1)	5,120,000	16.5 (14.9–18.2)	3,860,000	19.7 (18.4–21.1)	8,980,000
Some college (no degree)	19.8 (17.6–22.1)	3,420,000	18.1 (16.4–19.8)	3,370,000	18.9 (17.6–20.3)	6,790,000
Associate degree	17.1 (14.7–19.6)	1,990,000	16.4 (14.4–18.5)	2,330,000	16.8 (15.2–18.3)	4,330,000
Undergraduate degree	9.1 (7.7–10.5)	1,990,000	6.4 (5.4–7.5)	1,530,000	7.7 (6.8–8.6)	3,520,000
Graduate degree	5.5 (4.1–6.9)	730,000	3.5 (2.5–4.5)	510,000	4.5 (3.6–5.3)	1,250,000
**Poverty status^††^**						
At or above poverty level	16.4 (15.4–17.3)	16,380,000	12.3 (11.5–13.0)	12,650,000	14.3 (13.6–14.9)	29,030,000
Below poverty level	28.8 (25.8–31.9)	3,500,000	22.7 (20.4–25.0)	3,770,000	25.3 (23.4–27.2)	7,270,000
Unspecified	14.2 (10.9–17.5)	770,000	10.2 (7.5–12.8)	690,000	12.0 (9.8–14.1)	1,470,000
**U.S. Census region^§§^**						
Northeast	15.2 (13.3–17.0)	3,260,000	11.5 (9.9–13.1)	2,640,000	13.3 (11.9–14.6)	5,910,000
Midwest	19.2 (17.4–20.9)	4,950,000	17.8 (16.2–19.5)	5,050,000	18.5 (17.2–19.7)	10,000,000
South	19.7 (17.9–21.5)	8,310,000	14.2 (12.8–15.6)	6,370,000	16.9 (15.5–18.2)	14,680,000
West	14.6 (13.0–16.3)	4,120,000	10.1 (8.7–11.4)	3,030,000	12.3 (11.1–13.4)	7,160,000
**Health insurance coverage^¶¶^**						
Private insurance	13.5 (12.5–14.4)	10,490,000	10.1 (9.4–10.9)	8,170,000	11.8 (11.1–12.4)	18,670,000
Medicaid	27.7 (24.5–30.9)	3,260,000	23.9 (21.6–26.2)	4,650,000	25.3 (23.4–27.3)	7,910,000
Medicare only (≥65)	11.8 (9.4–14.2)	830,000	9.1 (7.4–10.8)	910,000	10.2 (8.8–11.7)	1,750,000
Other public insurance	21.9 (18.8–25.1)	1,540,000	17.1 (14.0–20.3)	970,000	19.8 (17.4–22.2)	2,510,000
Uninsured	32.8 (29.5–36.1)	4,270,000	22.6 (19.7–25.6)	2,250,000	28.4 (26.1–30.7)	6,530,000
Disability/Limitation***						
Yes	25.5 (22.8–28.2)	2,470,000	18.0 (16.1–20.0)	2,320,000	21.2 (19.6–22.9)	4,790,000
No	16.4 (15.3–17.6)	6,360,000	12.6 (11.6–13.6)	5,630,000	14.4 (13.6–15.2)	11,990,000
**Sexual orientation^†††^**						
Straight	17.3 (16.3–18.2)	19,230,000	13.5 (12.7–14.2)	15,920,000	15.3 (14.6–16.0)	35,160,000
Gay/Lesbian/Bisexual	23.8 (17.6–30.1)	620,000	17.9 (13.8–22.0)	600,000	20.5 (16.7–24.3)	1,230,000
**Serious psychological distress (Kessler Scale)^§§§^**						
Yes	39.3 (33.3–45.2)	1,290,000	33.6 (28.8–38.5)	1,720,000	35.8 (32.1–39.6)	3,010,000
No	16.8 (15.9–17.8)	18,610,000	12.7 (11.9–13.5)	14,850,000	14.7 (14.0–15.4)	33,460,000

**Abbreviations:** AI/AN = American Indian/Alaska Native; CI = confidence interval; GED = General Education Development certificate.

* Persons who reported smoking ≥100 cigarettes during their lifetime and who, at the time of interview, reported smoking every day or some days. Excludes 111 respondents whose smoking status was unknown.

^†^ Population estimates are calculated from extrapolated probability weights and are rounded down to the nearest 10,000 persons. Therefore, they may not add up to the overall population estimate.

^§^ Excludes 89 respondents of non-Hispanic unknown race. Unless otherwise indicated, all racial/ethnic groups are non-Hispanic; Hispanics can be of any race.

^¶^ Does not include Native Hawaiians or Other Pacific Islanders.

** Among persons aged ≥25 years. Excludes 107 persons whose education level was unknown.

^††^ Family income is reported by the family respondent who might or might not be the same as the sample adult respondent from whom smoking information is collected. 2016 estimates are based on reported family income and family size, based on the 2015 poverty thresholds published by the U.S. Census Bureau.

^§§^ *Northeast*: Connecticut, Maine, Massachusetts, New Hampshire, New Jersey, New York, Pennsylvania, Rhode Island, and Vermont; *Midwest*: Illinois, Indiana, Iowa, Kansas, Michigan, Minnesota, Missouri, Nebraska, North Dakota, Ohio, South Dakota, and Wisconsin; *South*: Alabama, Arkansas, Delaware, District of Columbia, Florida, Georgia, Kentucky, Louisiana, Maryland, Mississippi, North Carolina, Oklahoma, South Carolina, Tennessee, Texas, Virginia, and West Virginia; *West*: Alaska, Arizona, California, Colorado, Hawaii, Idaho, Montana, Nevada, New Mexico, Oregon, Utah, Washington, and Wyoming.

^¶¶^ *Private coverage*: Includes adults who had any comprehensive private insurance plan (including health maintenance organizations and preferred provider organizations). *Medicaid*: For adults aged <65 years, includes adults who do not have private coverage, but who have Medicaid or other state-sponsored health plans including Children's Health Insurance Program (CHIP). For adults aged ≥65 years, includes adults who do not have any private coverage but have Medicare and Medicaid or other state-sponsored health plans including CHIP; *Medicare only*: Includes older adults who only have Medicare coverage; *Other coverage*: Includes adults who do not have private insurance, Medicaid, or other public coverage, but who have any type of military coverage or Medicare (for those aged <65 years). This category also includes adults who are covered by other government programs. *Uninsured*: Includes adults who have not indicated that they are covered at the time of the interview under private health insurance, Medicare, Medicaid, CHIP, a state-sponsored health plan, other government programs, or military coverage.

*** Disability/limitation was defined based on self-reported presence of selected impairments including vision, hearing, cognition, and movement. Limitations in performing activities of daily living was defined based on response to the question, “Does [person] have difficulty dressing or bathing?” Limitations in performing instrumental activities of daily living was defined based on response to the question, “Because of a physical, mental, or emotional condition, does [person] have difficulty doing errands alone such as visiting a doctor’s office or shopping?” Any disability/limitation was defined as a “yes” response pertaining to at least one of the disabilities/limitations listed (e.g., vision, hearing, cognition, movement, activities of daily living, or instrumental activities of daily living). A random sample of half the respondents from the 2016 Person File were asked about disability/limitation. Disability/limitation estimates (%, population estimate) were obtained used the specific adult disability weight.

^†††^ Response options provided on the National Health Interview Survey were “straight, that is, not gay” for men, and “straight, that is, not gay or lesbian” for women.

^§§§^ The Kessler psychological distress scale is a series of six questions that ask about feelings of sadness, nervousness, restlessness, worthlessness, and feeling like everything is an effort in the past 30 days. Participants were asked to respond on a Likert Scale ranging between “None of the time” (score = 0) and “All of the time” (score = 4). Responses were summed over the six questions; any person with a score of ≥13 was coded as having serious psychological distress, and respondents with a score <13 were coded as not having serious psychological distress.

Among current smokers, the proportion of daily smokers was 76.1% in 2016, which declined from 2005 (80.8%, p-value for trend <0.05) (data not shown). Whereas mean number of cigarettes smoked per day declined from 2005 (16.7) to 2016 (14.1) among daily smokers (p-value for trend <0.05), no change occurred between 2015 (14.2) and 2016 (14.1) (data not shown). During 2005–2016, increases occurred in the proportion of daily smokers who smoked 1–9 cigarettes per day (from 16.4% to 25.0%) or 10–19 (from 36.0% to 39.0%) cigarettes per day (Figure 1). At the same time, decreases occurred in the proportion of daily smokers who smoked 20–29 (from 34.9% to 28.4%) or ≥30 (from 12.7% to 7.5%) cigarettes per day during 2005–2016 (p-value for trend <0.05). No significant changes in any category of number of cigarettes smoked per day occurred during 2015–2016.

**FIGURE 1:**
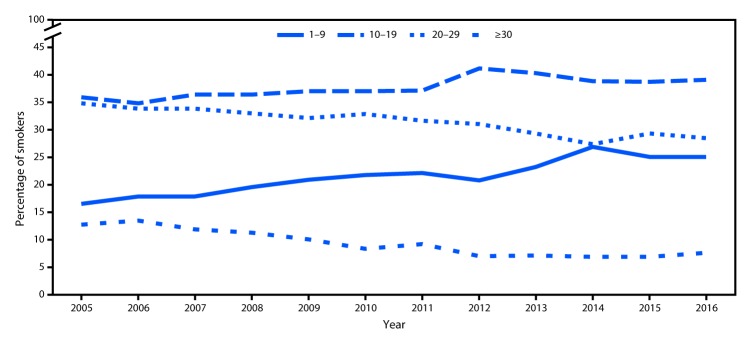
Percentage of daily smokers* aged ≥18 years who smoked 1–9, 10–19, 20–29, and ≥30 cigarettes per day — National Health Interview Survey, United States, 2005–2016 * Persons who had smoked ≥100 cigarettes during their lifetime and reported smoking cigarettes every day at the time of interview.

The overall quit ratio increased from 50.8% in 2005 to 59.0% in 2016 (p<0.05). During 2005–2016, the largest increase in quit ratios occurred among adults aged 25–44 years (from 37.0% to 48.9% [p<0.05]) (Figure 2).

**FIGURE 2:**
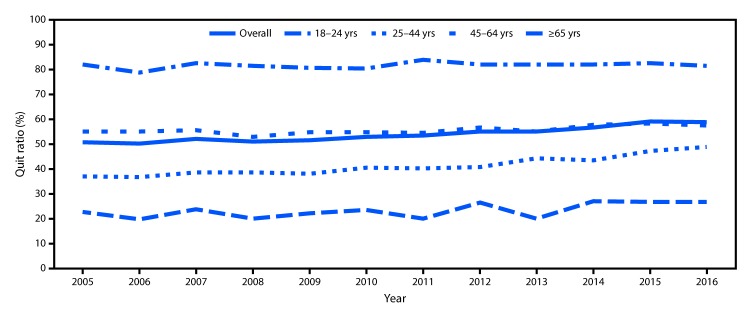
Quit ratios* among ever smokers^†^ aged ≥18 years, overall and by age group — National Health Interview Survey, United States, 2005–2016^§^ * Quit ratios defined as the ratio of former smokers to ever smokers for each survey year. ^†^ Respondents aged ≥18 years who reported having smoked ≥100 cigarettes during their lifetime. ^§^ p-value for trend 2005–2016 adjusted for sex and race/ethnicity: overall: p<0.0001; 18–24 years: p = 0.0064; 25–44 years: p<0.0001; 45–64 years: p = 0.0002; ≥65 years: p = 0.0874.

## Discussion

During 2005–2016, the prevalence of cigarette smoking among U.S. adults declined from 20.9% to 15.5%, and the proportion of ever smokers who had quit increased. However, during 2015–2016, cigarette smoking prevalence did not change significantly. In 2016, 37.8 million U.S. adults were current cigarette smokers, and marked sociodemographic differences in smoking prevalence persist. Proven population-based interventions, including tobacco price increases, comprehensive smoke-free laws, anti-tobacco mass media campaigns, and barrier-free access to tobacco cessation counseling and medications, are critical to reduce cigarette smoking and smoking-related disease and death among U.S. adults, particularly among subpopulations with the highest prevalences.

The observed disparities in smoking prevalence are likely attributable to multiple factors (*1*). Racial or ethnic differences might be partly explained by sociocultural influences and norms related to the acceptability of tobacco use and variations in exposure to tobacco marketing, whereas disparities by education might be partly attributable to variations in understanding of the range of health hazards caused by smoking (*3*,*4*). Variations in access to evidence-based tobacco cessation treatments through insurance coverage might partially explain the differences observed across insurance types (*5*). Smoking prevalence was higher among persons with severe psychological distress (*6*,*7*), potentially because of higher levels of addiction and dependence, lack of financial resources, less access to cessation treatments, and stressful living conditions among these persons (*6*,*7*). Assessing the smoking status of all patients served in psychiatric inpatient and outpatient settings, integrating evidence-based cessation interventions into mental health treatment plans, and implementing tobacco-free campus policies in mental health care facilities could help reduce smoking in this population (*6*,*7*).

During 2005–2016, an increasing proportion of adults who ever smoked cigarettes had quit smoking. However, following consecutive significant annual declines during 2013–2014 and 2014–2015 (*8*), no change in smoking prevalence was observed between 2015 and 2016. Moreover, longstanding declines in the proportion of daily smokers who smoked ≥20 cigarettes per day have stalled in recent years. These findings could be the result of multiple factors, including slowed progress in the adoption of proven interventions (*9*), or increased nicotine dependence from the concurrent use of other tobacco products (*1*). These findings underscore the importance of enhanced and sustained implementation of proven population-level interventions to continue previously observed annual declines in adult cigarette smoking (*2*).

The findings in this report are subject to at least five limitations. First, smoking status was self-reported and not validated by biochemical testing; however, self-reported smoking status is correlated with serum cotinine levels (*10*). Second, because NHIS does not include institutionalized populations and persons in the military, results are not generalizable to these groups. Third, the NHIS response rate of 54.3% might have resulted in nonresponse bias, even after adjustment for nonresponse. Fourth, the assessment of broad racial/ethnic populations (e.g., Asians and Hispanics) can mask differences in smoking prevalence among subgroups of these populations.^¶¶^ Finally, these estimates might differ from those reported from other surveys. These differences can be partially explained by varying survey methodologies and definitions of current smoking; however, trends in prevalence are comparable across surveys (*1*).

Sustained implementation of comprehensive state tobacco control programs can accelerate progress toward reducing adult smoking prevalence (*2*). Targeted interventions are warranted to reach subpopulations with the highest incidence of use, and can result in substantial reductions in tobacco-related disease and death and billions of dollars in savings from averted medical costs (*1*).

**Summary**

**What is already known about this topic?**

The U.S. Surgeon General has concluded that the burden of death and disease from tobacco use in the United States is overwhelmingly caused by cigarettes and other combusted tobacco products. Cigarettes are the most commonly used tobacco product among U.S. adults, and about 480,000 deaths per year are caused by cigarette smoking and secondhand smoke exposure.

**What is added by this report?**

The proportion of U.S. adults who smoke cigarettes declined from 20.9% in 2005 (45.1 million smokers) to 15.5% in 2016 (37.8 million smokers), but cigarette smoking prevalence did not change significantly during 2015–2016. Sociodemographic disparities in cigarette smoking persist. During 2005–2016, increases occurred in the proportion of adult ever smokers who quit smoking (50.8% to 59.0%).

**What are the implications for public health practice?**

Proven population-based interventions, including tobacco price increases, comprehensive smoke-free laws, high-impact anti-tobacco media campaigns, and barrier-free access to tobacco cessation counseling and medications, are critical to reducing cigarette smoking and smoking-related disease and death among U.S. adults, particularly among subpopulations with the highest smoking prevalence.

## Footnotes

* https://www.healthypeople.gov/2020/topics-objectives/topic/tobacco-use/objectives.

† Based on reported family income and family size: 2005 estimates are based on reported family income and family size, using the 2004 poverty thresholds published by the U.S. Census Bureau, and 2016 estimates are based on reported family income and family size, using the 2015 poverty thresholds published by the U.S. Census Bureau.

§ *Northeast:* Connecticut, Maine, Massachusetts, New Hampshire, New Jersey, New York, Pennsylvania, Rhode Island, and Vermont. *Midwest:* Illinois, Indiana, Iowa, Kansas, Michigan, Minnesota, Missouri, Nebraska, North Dakota, Ohio, South Dakota, and Wisconsin. *South:* Alabama, Arkansas, Delaware, District of Columbia, Florida, Georgia, Kentucky, Louisiana, Maryland, Mississippi, North Carolina, Oklahoma, South Carolina, Tennessee, Texas, Virginia, and West Virginia. *West:* Alaska, Arizona, California, Colorado, Hawaii, Idaho, Montana, Nevada, New Mexico, Oregon, Utah, Washington, and Wyoming.

¶ *Private coverage*: Includes adults who had any comprehensive private insurance plan (including health maintenance organizations and preferred provider organizations). *Medicaid*: For adults aged <65 years, includes adults who do not have private coverage, but who have Medicaid or other state-sponsored health plans including Children's Health Insurance Program (CHIP). For adults aged ≥65 years, includes adults who do not have any private coverage but have Medicare and Medicaid or other state-sponsored health plans including CHIP; *Medicare only*: Includes older adults who only have Medicare coverage; *Other coverage*: Includes adults who do not have private insurance, Medicaid, or other public coverage, but who have any type of military coverage or Medicare (for those aged <65 years). This category also includes adults who are covered by other government programs. *Uninsured*: Includes adults who have not indicated that they are covered at the time of the interview under private health insurance, Medicare, Medicaid, CHIP, a state-sponsored health plan, other government programs, or military coverage.

** Disability/limitation was defined based on self-reported presence of selected impairments including vision, hearing, cognition, and movement. Limitations in performing activities of daily living was defined based on response to the question, “Does [person] have difficulty dressing or bathing?” Limitations in performing instrumental activities of daily living was defined based on response to the question, “Because of a physical, mental, or emotional condition, does [person] have difficulty doing errands alone such as visiting a doctor’s office or shopping?” Any disability/limitation was defined as a “yes” response pertaining to at least one of the disabilities/limitations listed (e.g., vision, hearing, cognition, movement, activities of daily living, or instrumental activities of daily living). A random sample of half the respondents from the 2016 Person File were asked about disability/limitation. Disability/limitation estimates (%, population estimate) were obtained using the specific adult disability weight.

†† Starting in 2013, sexual orientation questions were added to NHIS for the first time. To determine sexual orientation, adult respondents were asked, “Which of the following best represents how you think of yourself?” with a response options of gay (“lesbian or gay” for female respondents), straight, that is, “not gay” (“not lesbian or gay” for female respondents), bisexual, something else, and I don’t know the answer.

§§ The six-question K6 scale was developed to identify persons with a high likelihood of having a diagnosable mental illness and associated functional limitations. The K6 scale asked how often during the past 30 days the respondents felt a) so sad that nothing could cheer them up; b) nervous; c) restless or fidgety; d) hopeless; e) that everything was an effort; and f) worthless. Responses were on a five-point Likert scale ranging from none of the time to all of the time. For each question, a value of zero, one, two, three, or four was assigned to the response “none of the time,” “a little of the time,” “some of the time,” “most of the time,” or “all of the time,” respectively. Responses to the six items were summed to yield a K6 score between 0 and 24, with a score of 13 or higher indicating serious psychological distress. Additional information available at https://www.cdc.gov/nchs/data/databriefs/db203.pdf.

¶¶ https://www.cdc.gov/mmwr/volumes/65/wr/mm6530a1.htm.
